# Controlling Factors of Soil CO_2_ Efflux in *Pinus yunnanensis* across Different Stand Ages

**DOI:** 10.1371/journal.pone.0127274

**Published:** 2015-05-21

**Authors:** Shaojun Wang, Jixia Zhao, Qibo Chen

**Affiliations:** Department of Environmental Science and Engineering, Southwest Forestry University, Kunming, Bailongshi, China; Tennessee State University, UNITED STATES

## Abstract

The characteristics of soil respiration (*Rs*) across different stand ages have not been well investigated. In this study, we identified temporal variation of *Rs* and its driving factors under three nature forest stands (e.g. 15-yr-old, 30-yr-old, and 45-yr-old) of *Pinus yunnanensis* in the Plateau of Mid-Yunnan, China. No consistent tendency was found on the change of *Rs* with the stand ages. *Rs* was ranked in the order of 30-yr-old > 45-yr-old >15-yr-old. *Rs* in 15-yr-old stand was the most sensitive to soil temperature (*Ts*) among the three sites. However, *Ts* only explained 30-40% of the seasonal dynamics of *Rs* at the site. Soil water content (*Sw*) was the major controlling factor of temporal variation at the three sites. *Sw* explained 88-93% of seasonal variations of *Rs* in the 30-yr-old stand, and 63.7-72.7% in the 15-yr-old and 79.1-79.6% in the 45-yr-old stands. In addition, we found that *pH*, available nitrogen (*AN*), C/N and total phosphorus (*TP*) contributed significantly to the seasonal variation of *Rs*. *Sw* was significantly related with *pH*, total nitrogen (*TN*), *AN* and *TP*, suggesting that *Sw* can affect *Rs* through improving soil acid-base property and soil texture, and increasing availability of soil nutrient. The results indicated that besides soil water, soil properties (e. g. *pH*, *AN*, C/N and *TP*) were also the important in controlling the temporal variations of *Rs* across different stand ages in the nature forestry.

## Introduction

Whether forest is a sink or source of atmospheric CO_2_ depends on the equilibrium between two large fluxes of photosynthesis and respiration. Soil respiration (*R*_*S*_) is a primary path through which CO_2_ fixed by photosynthesis returns to the atmosphere [1, 2]. A slight fluctuation in soil respiration can induce a large change in global carbon cycle. Therefore, *Rs* may have a significant effect on the CO_2_ sink of forest ecosystems and the future balance of atmospheric CO_2_ [3, 4].

Considerable interests were focused on the balance and deposition of soil C in forest ecosystems [5], especially on the seasonal variations of soil CO_2_ efflux across different stand ages [6]. The effects of stand ages on soil respiration varied across the different studies. *Rs* was reported to decrease with stand age in temperate forests and increase with stand age in tropical and subtropical forests [7, 8]. Soil respiration may differ as abiotic and biotic factors fluctuate across different stand ages [9, 10]**.**

Soil temperature (*Ts*) is a major factor controlling soil respiration because of the effect on microbial decomposition in soil and root respiration in ecosystem [11]. *Rs* is widely proved to be markedly sensitive to soil temperature [12, 13]. The sensitivity of *Rs* to soil temperature is usually assessed by temperature coefficient (*Q*_*10*_). *Q*_*10*_ represents the factor by which *R*_*S*_ increases with every increment of 10°C The *Q*_*10*_-based model is often used to calculate *Rs* from local to global scales [14, 15, 16].

Soil water deficit can restrain the positive effect of *Ts* on *Rs* [17–20]. Reduced *Sw* under drought conditions suppresses soil microbial activity regardless of soil temperature, and also decreases the temperature sensitivity of *Rs* [21]. *Rs* and its temperature sensitivity (*Q*_*10*_) decreased sharply when *Sw* dropped below 0.15 m^3^ m^-3^ [22]. The interactions of temperature-water can explain most seasonal variation of CO_2_ efflux. However, they contribute to the temperature effect on *Rs* only when *Sw* is sufficient to permit significant root production and microbial respiration [23]. In recent years, Yunnan experienced the severe droughts, highlighting future climate threats on forest ecosystem [24]. Severe drought influenced *Sw*, plant root dynamics, litter fall, soil organic matter and nutrient mineralization, which in turn affected *Rs* processes [25].

Soil factors (e. g. substrate supply, soil organic matter, soil texture and soil *pH*) have important effects on soil respiration, while soil temperature together with soil water content are the main factors controlling the variation of soil CO_2_ efflux [26, 27]. Predicting temporal variation of *Rs* and its response to climate change requires a thorough understanding of the dependence of *Rs* processes on these environment variables.

*Pinus yunnanensis* is one of the main forest types in yunnan-guizhou plateau region, accounting for about 70% of forest area in the Yunnan province. The aim of the present work is to advance in the understanding of soil respiration dynamics and its controlling factors under the three stand ages. The specific objectives of this study are: (1) to examine whether soil respiration differs among stand ages and (2) to determine the temporal variation of *Rs* and its relationship to some possible driving variables (e. g. soil temperature, soil water, soil pool size of C, N, and *pH*) in the *Pinus yunnanensis* nature forest of southwestern China.

## Materials and Methods

### Ethics Statement

The management ownership of study sites belongs to Southwest Forestry University. No specific permit was required for our study, because the work didn't involve any endangered or protected species, and didn't do harm to environment.

### Site description

The study was conducted in the Millstones Mountain National Forest Park in Yunnan Province (101°16′06″, 23°46′18″). The sites (Yuxi of Yunnan Forest Ecosystem Positioning Research Station) are located in geographical comprehensive department of the Yunnan-Guizhou plateau and the southern margin of Qinghai-Tibet plateau. The area belongs to a subtropical/typical mountain climate region. Annual mean temperature is about 15°C and annual rainfall is about 1050 mm. Precipitation shows a strong seasonal variation. About 85% rainfall is in a rainy season (from May to October), and only 15% rainfall is in a dry season (from November to April of next year).

Three sites with different stand ages (e. g. 15-yr-old, 30-yr-old, and 45-yr-old) in the nature forestry of *Pinus yunnanensis* were established to determine the effects of stand age on soil respiration. The three sites (850 m apart) had same parent material (basalt), similar altitude (less than 50 m altitude difference), similar initial conditions of soil and succession. Their characteristics were briefly summarized in Table 1.

**Table 1:** Site conditions at the three sites in the Millstones Mountain National Forest Park in Yunnan Province.

Sites	Elevation (m)	Stem density(trees ha^-1^)	Leaf area index (m^2^m^-2^)	Soil types	Dominant species	Litter layer thickness (cm)	Humus layer thickness (cm)	Average DBH (cm)	Average Height (m)	Canopy coverage (%)
15-yr old	2180	1250	8.4	Red soil	*Pinus yunnanensis*, *Vaccinium fragile*, *Vaccinium bracteatum*, *Fargesia spathacea*	1–2	5	8	6.5	55
30-yr old	2178	1625	11.5	Red soil	*Pinus yunnanensis*, *Quercus aliena*, *Schima superba*	5–7	12	13	10.3	90
45-yr old	2240	900	7.3	Red soil	*Pinus yunnanensis*, *Quercus aliena*, *Keteleeria evelyniana*, *Vaccinium fragile*	3–4	8	25	14.2	75

### Measurements of soil respiration and soil properties

Three measuring plots (30 × 15 m) were randomly selected at the each site in the nature forestry of *Pinus yunnanensis*, and 4 measurements in each plot were carried on the soil respiration and soil properties (e. g. soil temperature, soil water content, *pH*, soil organic matter, total soil nitrogen, and soil available nitrogen). At the three sites, *Rs* was measured in the dry seasons (Apr and Dec in 2012, and Mar 2013) and in the wet seasons (Jul and Oct 2012), as the climate characterized by less change of air temperature and strong wet-dry variation. *Rs* was monitored around the 20th day of each measurement. *Rs* was measured between 10:00 and 16:00 hours in a small PVC collar (10 cm in diameter and 5 cm in height) installed 2–3 cm into the soil 2 weeks in advance. All ground vegetation within the collars was regularly removed by clipping to avoid interference of respiration from plants. We used the Li 6000–09 soil respiration chamber (LiCor Inc, Lincoln, NE, USA) in which the efflux of CO_2_ concentration was recorded with Li 6250 infrared gas analyzer (LiCor Inc). Soil temperature was monitored by a thermocouple penetration probe (Li6000-09 TC, LiCor Inc) inserted in the soil to a depth of 5cm in the vicinity of soil respiration chamber, while the soil CO_2_ efflux was measured. This work was conducted based on Forestry Standards “Observation Methodology for Long-term Forest Ecosystem Research” of People’s Republic of China (LY/T 1952–2011).

Soil cores at the sites were collected in the positions of PVC collar to analyze soil properties after measuring of *Rs*. *Sw* at depths of 0–5 cm was determined gravimetrically after drying approximately 20 g of fresh soil at 105°C for 48 h. Soil organic matter (*SOM*) was determined by dichromate oxidation with external heating procedure, total N (*TN*) by Kjeldahl digestion method, and soil available nitrogen (*AN*) by alkaline hydrolysis diffusion method. Soil *pH* was measured with direct potentiometry, and total phosphorus (*TP*) with colorimetric method [28].

### Calculation and data analysis

The functions of exponential regression (Van’t Hoff Eq (1)), and nonlinear regression (Arrhenius Eq (2)), and Lloyd and Taylor Eq (3)) [29, 30] were used to fit the relationship between *Rs* and soil temperature. We also performed linear, power and quadratic regression analyses of *Rs* against *Sw* using Eq (4) as follows:

$$R_{S}=\text{a}{\text{e}}^{\text{b}T}{{}_{}}_{,}\;Q_{10}={\text{e}}^{10\text{b}}$$ (1)

$$R_{S}=\text{a}{\text{e}}^{-\text{E}\;/\;\text{R}(T+273.2)}$$ (2)

$$R_{S}=R_{ref}{\text{e}}^{E_{0}(1/Tre\text{f}\;\text{-}1\;/\;T\text{-}T_{0})}$$ (3)

$$\text{Linear}:R_{S}=\text{a}+\text{b}Sw,\;\text{Quadratic}:\;R_{S}=\text{a}+\text{b}Sw+\text{c}Sw^{2}\;\text{or Exponential}:R_{S}=\text{a}Sw^{\text{b}}$$ (4)

where a and b are fitted parameters, whereas *Q*_*10*_, E and R are temperature sensitivity of *Rs*, fitted apparent activation energy (J mol^-1^), and universal gas constant (8.134J mol^-1^ k^-1^), respectively. *R*_*ref*_ (μmol m^-2^ s^-1^ **)** and *T*_*ref*_ are the soil respiration and temperature under standard conditions. E_0_ and T_0_ are the activation-energy-type parameter and the lower temperature limit for *R*_*S*_, respectively. Next, the following linear and nonlinear models (Eqs (5)–(7)) were used to express the relationships among *Rs*, *Ts* and *Sw* (a, b and c are fitted constants):

$$R_{S}=\text{a}+\text{b}\left(T\;Sw\right)$$ (5)

$$R_{S}=\text{a}+\text{b}T+\text{c}\;Sw$$ (6)

$$R_{S}=\text{a}\;{\text{e}}^{\text{b}T}Sw^{\text{c}}$$ (7)

All statistical nonlinear regression and significant difference analyses were performed using SPSS 17.0 (SPSS for windows, Chicago, IL). All the data normality and equal variance were tested. Analysis of variance (ANOVA) was used to test the differences in *Rs*, *Ts* and *Sw* among the three sites. Regression analysis was applied to describe the relationships between *Rs*, and *Sw* and *Ts*. Pearson's correlation coefficients were used to express the relationships between *Rs* and soil properties (e. g. *pH*, soil organic matter, total soil nitrogen and soil available nitrogen).

## Results

### Temporal variations of *R*_*S*_, *Ts* and *Sw*

**Fig 1:**
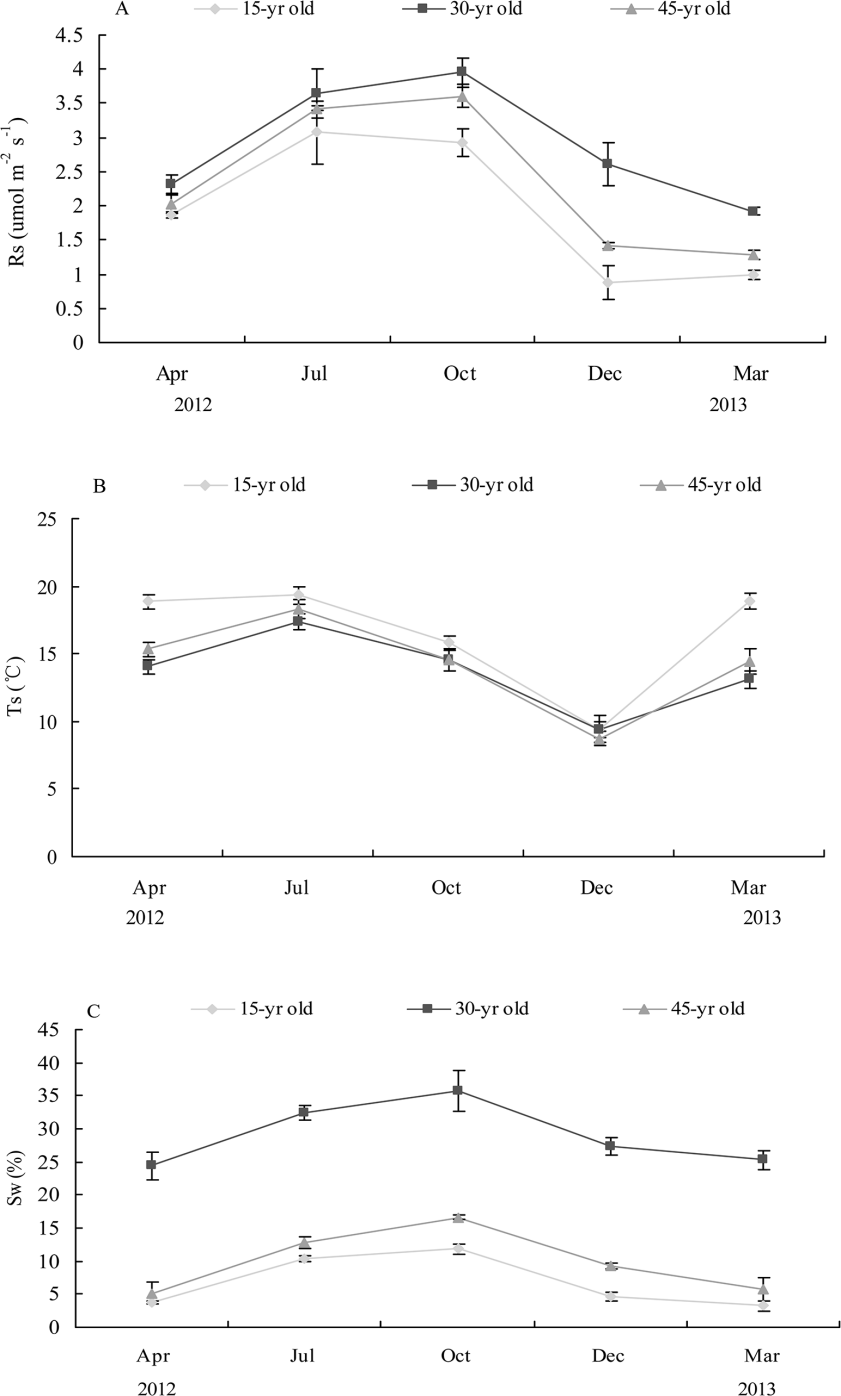
Seasonal variations of soil CO_2_ efflux (*R*_*S*_) (A), soil temperature (*T*_*S*_) (B), soil water content (*S*_*w*_) (C) at the 5 cm depths in different stand ages.

The temporal variations of *Rs* in the 30- and 45-yr-old stands were characterized by having the highest values in October and the lowest values in March, which followed the temporal dynamics of *Sw* (Fig 1 A and 1 C). However in the 15-yr-old stand, the maximum values of *Rs* occurred in July and the lowest point was in December, in accordance with the seasonal dynamics of *Ts* (Fig 1 A and 1 B). *Rs* was significant difference across the seasons at the three sites (*F* = 14.548, *p*<0.001). In the 30-year-old stand where *Sw* was the highest, *Rs* was significantly higher than that in the 15- and 45-yr-old stands (Fig 1).

Temporal variations of *Ts* weren't significantly different among the sites (*F* = 6.182, *p*>0.05) (Fig 1B). The low values of *Ts* were observed in autumn (December) and the highest values occurred in the summer (July). Soil water content (*Sw*) at 5 cm soil layer had a dry-wet cycle with the maximum in October, and the minimum in March or April (Fig 1C). There were significant differences in *Sw* among the sites (*F* = 10.315, *p*<0.05). *Sw* was higher in the 30-year-old stand than in the 15- and 45-yr-old stands (Fig 1C).

### Relationship between *Ts* and *R*_*S*_

Soil respiration (*Rs*) was significantly related with soil temperature (*Ts*) at these sites (Table 2). The Van’t Hoff and Arrhenius models showed the best fit between *Rs* and *Ts*, having the highest *R* ^*2*^. *Ts* can explain 27.8–39.7% of the seasonal changes of *Rs*, using Van’t Hoff. By contrast, *Ts* explained 27.1–40.2% of the seasonal change when Arrhenius function was used (Table 2).

**Table 2:** Regression analyses of *R*_*S*_ and *Q*_*10*_ against soil temperature at 5cm soil depth at the three sites.

Sites	*R*_*S*_ = ae^b*T*^				*R*_*S*_ = ae^-*E* / R(*T*+273.2)^				*R*_*S*_ = *R*_*ref*_ e^*E*^_*0*_^(1/*Tre*f-1 / *T*-*T*^_*0*_^)^		
	*a*	*b*	*R* ^*2*^	*Q*_*10*_	*a*	*E*	*R* ^*2*^	*Q*_*10*_	*E*_*0*_	*R* ^*2*^	*Q*_*10*_
15-yr old	0.480	0.074	0.397^**^	2.10	2404.548	19932.56	0.402^**^	1.68	343.12	0.394^**^	1.83
30-yr old	1.277	0.048	0.278 ^*^	1.62	3796.515	21200.55	0.271 ^*^	1.32	416.66	0.267^*^	1.64
45-yr old	1.172	0.042	0.285^*^	1.52	6416.267	23193.90	0.273^*^	1.36	521.34	0.261^*^	1.48

^*^ *P* < 0.05

^**^ *P* < 0.01.

*Rs* was more sensitive to *Ts* in the 15-yr-old stand than in the 30- and 45-yr-old stands (Table 2). The temperature sensitivity of *Rs* (*Q*_*10*_) varied among stand ages. The *Q*_*10*_ values ranged from 1.52 to 2.10 with the Van't Hoff regression. In contrast, *Q*_*10*_ values (from 1.32 to 1.68) were the lowest among the sites, using the Arrhenius function (Table 2).

### Relationship between soil water and *R*_*S*_

**Fig 2:**
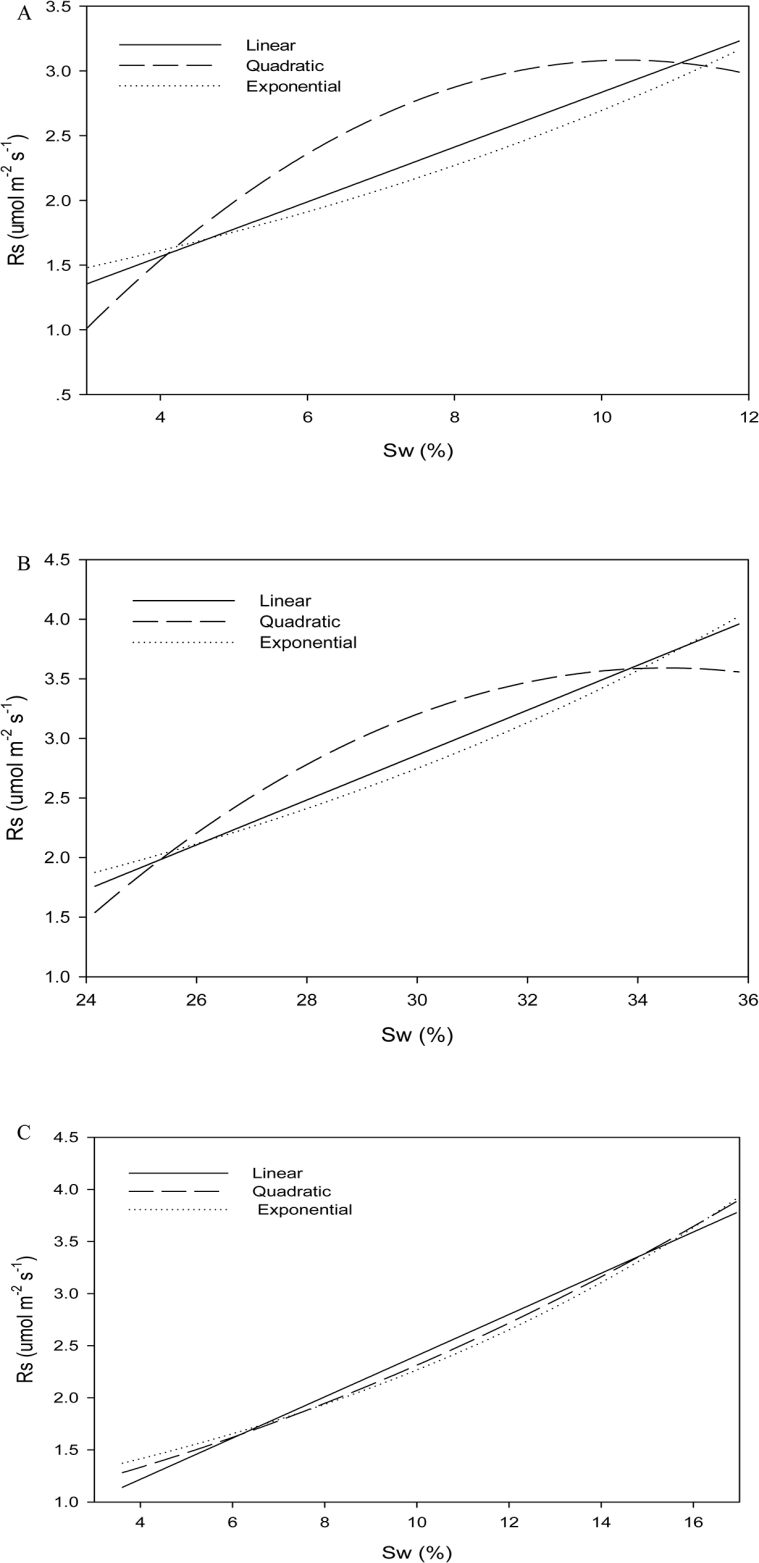
Regression analyses of soil respiration (*R*_*S*_) against soil water content (*Sw*) at 5cm soil depth in the 15- yr-old stand (A), 30- yr-old stand (B), and 45- yr-old stand (C).

The regression analyses were conducted using linear, power and quadratic models to quantify the relationship between *Rs* and *Sw* (Table 3 and Fig 2). The correlations between *Rs* and *Sw* were significant, and quadratic models fitted the best at the sites (Table 3). *Sw* explained 88–93% of the seasonal changes of *Rs* in the 30-yr-old stand, while it explained 63.7–72.7% in 15-yr-old, and 79.1–79.6% in 45-yr-old stand.

**Table 3:** Regression analyses of *R*_*S*_ against *Sw* at 5cm soil depth in the three sites.

Sites	*R*_*S*_ = a+b*Sw*			*R*_*S*_ = a+b*Sw* + c*Sw* ^2^					*R*_*S*_ = a*Sw* ^b^ ${\text{R}}_{\text{S}}={\text{R}}_{\text{ref}}{\text{e}}^{{\text{E}}_{0}\left(\frac{1}{{\text{T}}_{\text{ref}}-{\text{T}}_{0}}-\frac{1}{\text{T}-{\text{T}}_{0}}\right)}$			
	*a*	*b*	*R* ^2^	*a*		*b*	*c*	*R* ^2^	*a*		*b*	*R* ^2^
15-yr old	0.719	0.212	0.669^*^		-1.036	0.798	-0.039	0.727^*^		1.143	0.086	0.637^*^	
30-yr old	-2.793	0.189	0.902^**^		-19.264	1.325	-0.019	0.930^**^		0.386	0.065	0.880^**^	
45-yr old	0.427	0.198	0.792^**^		0.876	0.095	0.005	0.796^**^		1.033	0.079	0.791^**^	

^*^ *P* < 0.05

^**^ *P* < 0.01.

The temperature-based model represents the relationship between *Rs* and *Ts*. However, it cannot account for the influence of *Sw* (Table 2). Therefore, we integrated both *Ts* and *Sw* into three equations (Eqs 5–7) to model the combined effects of *Ts*, *Sw* on *Rs* (Table 4). In comparison with the one-dimensional equation above, the *R* ^*2*^ of the two-dimensional equation increased with three models.

**Table 4:** Regression analyses of *R*_*S*_ against *Sw* and *Ts* at 5cm soil depth in the three sites.

Sites	*R*_*S*_ = a+b(*T Sw*)			*R*_*S*_ = a+b*T*+c *Sw*				*R*_*S*_ = a e^b*T*^ *Sw* ^C^			
	*a*	*b*	*R* ^2^	*a*	*b*	*c*	*R* ^2^	*a*	*b*	*c*	*R* ^2^
15-yr old	0.526	1.556	0.716^**^	4.375	0.091	0.808	0.869^**^	-1.429	0.134	22.159	0.762^**^
30-yr old	0.342	1.247	0.924^**^	2.526	0.075	0.642	0.953^**^	-3.114	0.187	17.346	0.902^**^
45-yr old	0.400	1.399	0.870^**^	3.658	0.083	0.715	0.892^**^	-2.019	0.176	19.139	0.815^**^

^*^ *P* < 0.05

^**^ *P* < 0.01.

### Relationships between soil properties and *R*_*S*_

**Fig 3:**
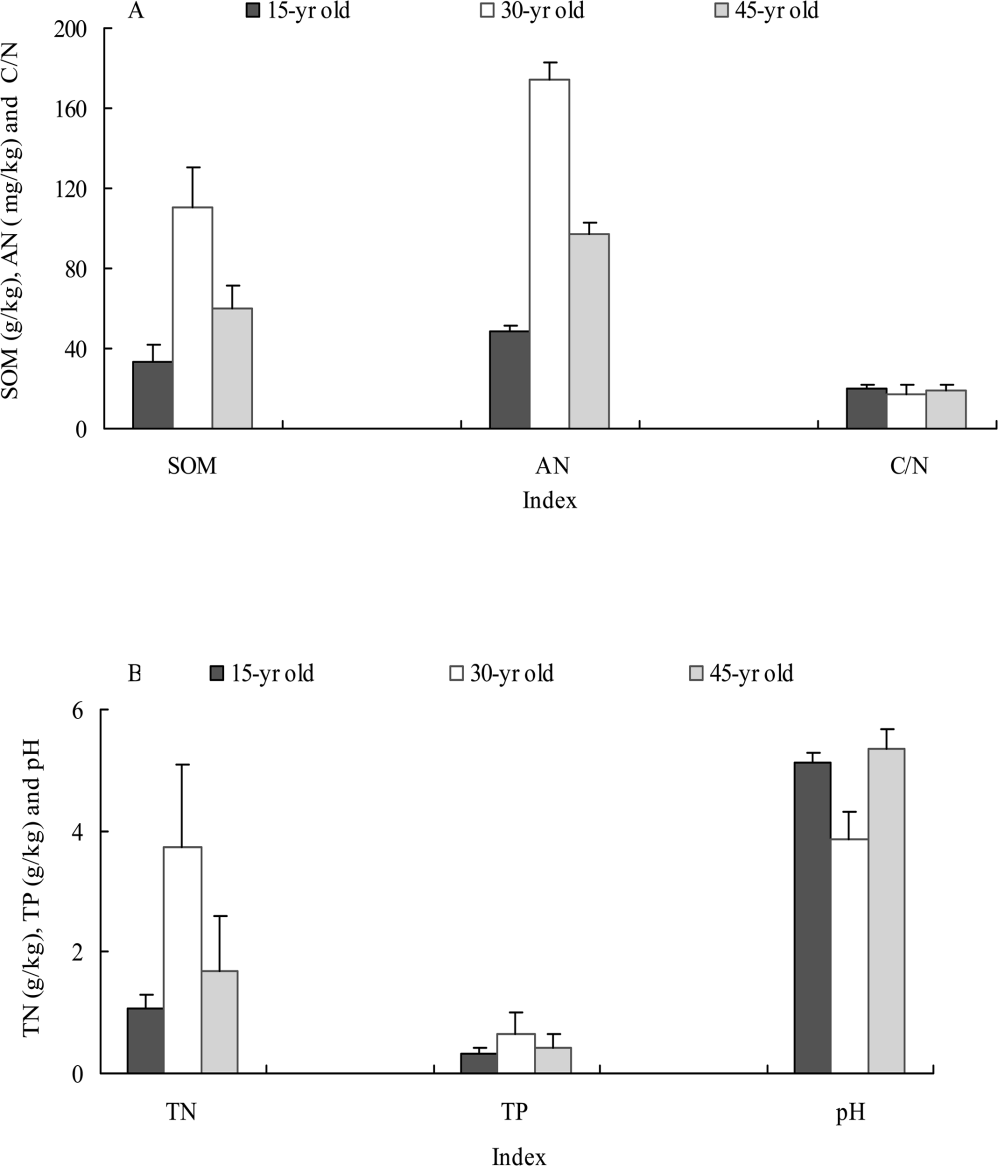
Soil physicochemical characteristics (A: *SOM*, *AN* and C/N; B: *TN*, *TP* and *pH*) across the three stand ages.

The significant correlations were detected among *Rs*, *Ts* and *Sw*, which explain much of the temporal variation of *Rs* at the sites of 15-, 30-, and 45-yr-old. We also identified the correlations between some soil physicochemical properties and seasonal variation of *Rs*. *Rs* in the 30- yr-old stand was the highest, which coincided with higher soil C and N among the three sites (Fig 3). *Rs* was also found to be positively correlated with *pH* (*p*<0.05), *AN* (*p*<0.05) and *TP* (*p*<0.01) at the three sites (Table 5). In contrast, negative correlation was detected between *Rs* and C/N (*p*<0.01). No significant correlations were found between the mean *Rs*, and *SOM* and *TN*. *Sw* was significantly related with *pH*, *TN*, *AN* and *TP*, suggesting that higher *Sw* may pay crucial influences on *R*_*S*_ through its influences on these soil physicochemical properties.

**Table 5:** Relationships between *Rs* and some soil properties in the three sites.

Sites	Item	*SOM* (g kg^-1^)	*TN* (g kg^-1^)	*AN* (mg kg^-1^)	*TP* (g kg^-1^)	*C/N*	*pH*
15-yr old	*Sw* (%)	0.314	0.905^**^	0.726^*^	0.713^*^	-0.698^*^	-0.02
	*Rs* (μmol CO_2_ m^-2^ s^-1^)	0.221	0.574	0.737^*^	0.953^**^	-0.933^**^	0.718^*^
30-yr old	*Sw* (%)	-0.116	0.724^**^	0.902^**^	0.872^**^	-0.782^**^	0.543^*^
	*Rs* (μmol CO_2_ m^-2^ s^-1^)	0.546	0.431	0.913^**^	0.890^**^	-0.901^**^	0.733^*^
45-yr old	*Sw* (%)	-0.136	0.702^**^	0.836^**^	0.893^**^	-0.674^**^	0.582^*^
	*Rs* (μmol CO_2_ m^-2^ s^-1^)	0.515	0.455	0.871^**^	0.890^**^	-0.921^**^	0.653^*^

^*^ *P* < 0.05

^**^ *P* < 0.01.

## Discussion

### Influence of *Sw* on *R*_*S*_ across different stand ages

Soil temperature and soil water are considered as main factors in controlling temporal variation of *R*_*S*_ [31, 32]. In the study, the variation of *R*_*S*_ in the 15-yr-old stand was in accordance with *Ts*. *Ts* at the site was the highest among the three stands and it only explained 30–40% of the seasonal dynamics of *Rs*. In contrast, *Sw* explained above 60% of *Rs* variations and the explained amount was greater than that explained by *Ts*. Meanwhile, the variation of *Rs* coordinated well with the temporal dynamics of *Sw* in the 30- and 45-yr-old stands. In the 30-yr-old stand, there was higher *Sw* as the larger canopy coverage and thicker litter layer can hold more soil water content, so *Rs* was significantly higher in the stand than in the 15- and 45-yr-old stands. The explained amount of *Sw* to seasonal changes of *Rs* was greater in the 30-yr-old stand than in the 15- and 45-yr-old stands. Therefore, *Sw* varied across different stand ages, which in turn exerted crucial effect on the temporal variability of *Rs* [33, 34].

In recent years, Yunnan has experienced severe droughts [24]. *Sw* is so low that the vitality of root and microorganism are suppressed. Therefore, *Rs* may not be promoted at the higher temperatures when soil moisture values were lower [35]. The limiting effect of *Sw* on *R*_*S*_ is a feature well documented in forest ecosystems [16, 17]. In this study, soil respirations were higher in wet seasons than in dry seasons, which was similar to the results reported in Ailao Mountains [36]. *R*_*S*_ was strongly influenced by *Sw* when *Sw* dropped below 10%. In addition, the maximum of *R*_*S*_ often occurred in Oct, when *Sw* was in its maximum. Therefore, soil water availability was important in controlling temporal variation of *Rs* among the three sites.

*R*_*S*_ in maximum often occur at intermediate moisture levels, and moisture functions are explained by some biogeochemical models. *Sw* below a threshold imposes desiccation stress on microbial decomposers. This can limit the diffusion of soluble substrates that are necessary for microbial respiration [37]. The decrease in *R*_*S*_ can also be explained by the changes in soil structural properties during drought, furthering the effect on soil microbes, the mobility of enzymes and substrates. Soil properties such as water repellency and aggregate structure can change with soil drying, affecting soil water holding capacity and surface tension [38, 39]. Water repellency induced by prolonged drying prevents the homogenous rewetting of the organic horizon, which delays the recovery of soil respiration [40]. *Sw* can affect the water-holding capacity of soil through increasing soil nutrient, improving soil construct, receding soil bulk density and enhancing soil porosity [41]. In the study, *Sw* was significantly related with *pH*, *TN*, *AN* and *TP*, thus higher *Sw* can pay crucial influences on *R*_*S*_ through the effects on these soil properties. Therefore, these results are important for the contexts of less frequent rainfall or increasing drought in forest ecosystems [42, 43].

### Influences of soil properties on *R*_*S*_ across different stand ages

There are some disagreements about the changes in soil respiration with stand age. Saiz et al. (2006) showed that *R*_*S*_ decreased with stand age [44]. By contrast, *R*_*S*_ was reported to increase with stand in a loblolly pine chronosequence [45]. These disaccords may be attributed to the differences in aboveground plant and some soil properties among stand ages, besides soil temperature and soil water.

Soil physicochemical characters (e.g. *SOM*, *TN*, *AN* and *TP*) fluctuated across stand ages. These parameters values were significantly higher in the 30-yr-old stand than in the 15- and 45-yr-old stands, which coincided well with the higher *Rs* among the sites. Furthermore, soil *pH*, *AN* and *TP* in the three sites were positively related with the seasonal variation of *Rs*, and C/N was negatively correlated with *Rs*. Soil *pH* can effect the variation of *Rs* through directly affecting on the tolerance of bacterial community, as biological activity of soil microorganisms is often permitted soil *pH* between a minimum of 3 and a maximum of 7 to 8 [46]. The correlations between *Rs* and *AN* may be explained by the dependence of plant growth and root activities on soil N availability [47]. Soil P availability increases the rate of soil CO_2_ efflux, through an increase in stem growth of trees [48]. Soil C/N showed a negative correlation with *Rs*, as low C/N can increase the microbial decomposition [49]. Finally, it is widely accepted that there is a positive correlation between plant productivity and soil respiration [50, 51]. In the study, there were greater leaf area index and canopy coverage, and thicker litter layer in the 30-yr-old stand, which can contribute to higher *R*_*S*_ at the site.
